# Chian Turpentine, and Its Use in Cancer

**Published:** 1880-12

**Authors:** 


### CHIAN TURPENTINE, AND ITS USE IN CANCER.

We clip the following abstract from a paper published in the *Lancet*, of October, 1880, by Dr. Jno. Gray, Obstetric Surgeon to the Queen's Hospital, Birmingham:

The results of the treatment of various forms of cancer, in the hands of certain medical practitioners, are alleged to be so different from those obtained by myself, that these gentlemen have thought fit to question the validity of my experiments. Certain results obtained by the use of a remedy, almost forgotten by the therapeutist, were placed by me unreservedly before the profession; and for the benefit of suffering humanity at least a fair trial of the remedy ought to be given before condemning it. In testing the efficiency of the drug, it is doubtless all-important that the true one should be used. It would appear to be not an easy matter to decide the question of purity, for upwards of three hundred specimens have been sent to me for examination by English and foreign medical men, most of them being guaranteed by the druggists supplying them, and it was a surprise to me to find so few examples of the genuine article amongst them. It will be as necessary now as before to use extreme vigilance in this respect in expectation of the new supply. In a large percentage of cases the factitions turpentine only has been used in the treatment of cancer. In one institution this was particularly so; and after the genuine drug had been tried for about three weeks the results of the two were combined, and a letter forthwith appeared in the *Times*, alleging that the remedy was useless. A similar instance occurred in an institution in this town, where the impure drug was first used, and then after a trial with the pure drug for about three weeks, letters appeared in a medical journal declaring that the remedy was of no benefit in the treatment of cancer. It is plain that sufficient time has not been allowed to elapse for the full effects of the remedy to be manifested, as in these instances of condemnation it has almost invariably been declared inoperative after a few weeks' trial only. Besides the unsatisfactory results which have accrued from the use of the impure drug, the full effects of the pure turpentine have not been gained in some instances from the drug not having been entirely digested. This has arisen occasionally from its incomplete preparation for administration. In compounding the mixture, freshly prepared mucilage of tragacanth, B. P., has not been used, and then it rapidly decomposes ; or sufficient of the mucilage has not been employed, which causes the resin to separate, and the doses of the medicine, therefore, have been irregular and insufficient. It has been shown in the *Pharmaceutical Journal* that acacia powder might be advantageously substituted for the tragacanth. It is necessary, therefore, to provide that a sufficient quantity of either gum shall be used in the preparation of the Chio turpentine mixture. Where the digestive powers are impaired, an addition of twenty minims of the compound tincture of cinchona to each dose has occasionally proved beneficial. The discontinuance of the sulphur has been advised, in order to make the mixture more elegant and miscible. I believe, however, that the mixture is more efficient, when it is well tolerated, if prepared with the sulphur.

In reference to the pills, if they are not properly prepared they are not always digested. If this is discovered on examining the evacuations, the use of the mixture in conjunction with the pills is indicated; that is, a dose of the mixture should be taken by the patient night and morning, and two pills in the middle of the day, in each case after food. It is advisable, when commencing the treatment, to give only half doses of the turpentine for the first week. Magnesia should not be used as an excipient in the preparation of the pills, but whether this exception ought to apply to the recently collected drug future experiment will have to decide. Pills prepared with magnesia are almost insoluble, and the same remark applies to some coated ones. The pills may be prepared sometimes with ether, to soften the turpentine, or it may be made soft by immersing the mortar in a hot water bath, the sulphur being added to the mass in its softened state. The latter mode appears preferable, if only to facilitate the digestion of the pills, as if any difficulty is met with in this way they may be roughly powdered and taken in milk or some bland liquid, and then they are generally digested.

The statements made in my original paper as to the efficacy of Chian turpentine in cancer appear to me to be fully confirmed by the additional experience gained in the use of the remedy. In the cases previously reported the cancer has disappeared, and there are no signs of its return. This fact must be considered at least as a complete refutation of the statements, recently and boldly made, that Chian turpentine in the treatment of cancer is useless. The cases just referred to are unique of their kind, for, so far as I know, they are the only instances on record of cancer being cured solely by the administration of an internal remedy. I feel justified, therefore, in stating that uterine cancer at least may be removed by the use of Chian turpentine.

It is a fact which might be expected that most of the cases of cancer which have presented themselves are in an advanced stage of the disease; and more especially is this the case in cancers of the uterus. Yet the treatment continues to be very efficient. My experience does not stand alone in this respect, for several gentlemen have given independent testimony of the good effects produced by the turpentine treatment in cancer of various organs. In my original experiments the turpentine alone was used in order to thoroughly test its efficacy, but it now appears that the use of certain local and general measures undoubtedly promotes the convalescence of the patient. It is possible, therefore, to formulate a line of treatment which shall be successful in cancers of the uterus. Hence, given a case of cancer limited to that organ, and a steady use of the remedy for six or eight months, continued with certain general and local remedies if necessary, and a disappearance of the cancer may be reasonably expected. Eleven cases of uterine cancer under this treatment at the Queen's Hospital, besides a number of others in private practice, so far justify the preceding statements. Some very interesting facts have been observed during their treatment, which I hope to detail on a future occasion.

It is advisable not to be too precipitate in rejecting a case of uterine cancer on the ground of its being too far advanced for treatment. The following case, amongst others, is to the point: Mary B. took her discharge from the Middlesex Hospital, London, about four months ago, and, according to report, was informed that her case was incurable, and that she had not long to live. She had heard of the turpentine treatment, and was very anxious to be under my care at the Queen's Hospital, but I advised her not to undertake the journey, as I believed the case, from what I heard of it, was too far advanced for treatment. This advice, however, she disregarded, and became an in-patient at the Queen's Hospital. Here she was placed under the Chian turpentine treatment, and improved so much in nine weeks that she is about to return to London with every prospect of soon being cured.

In cancer of the uterus the conclusion expressed in a former paper, that the remedy destroys the cancer cells and causes the death of the growth, is strengthened by experience, and, as a consequence, there is cessation of pain and hemorrhage in the first instance. But the turpentine at first does not destroy the bloodvessels of the part. As the dead tissue escapes, these become denuded, and give rise to hemorrhage, especially at the menstrual period. In patients who have past the climacteric period this has not been observed. Ergot has been found of no benefit in arresting the hemorrhage. Insufflation with three grains each of sulphate of zinc and powdered charcoal, once or twice a day, after syringing the vagina, has proved effectual, and appears to promote the obliteration and atrophy of the vessels. The same end may be attained by using a weak solution of perchloride of iron and glycerine. I have ordered some patients to syringe the vagina daily with equal parts of common vinegar and water, with good effect. When the anterior walls of the vagina are involved, either primarily or from extension, the case is not so suitable for treatment with the turpentine. A vesico-vaginal fistula is soon generally formed, but if this do not happen, crystitis supervenes, and the patient succumbs to the uræmic symptoms which are rapidly developed. Moreover, the turpentine is not well tolerated, but when a fistula forms, it is better borne, and uræmia does not arise.

Excision of the cervix uteri is not advisable, as the disease readily extends to the anterior wa l of the vagina subsequently; and then the bladder complications, above described, rise with increased force. The use of the curette is not so objectionable if it is employed to remove only the sloughing mass after some weeks' use of the China turpentine, but it has not been deemed necessary to use the instrument, it being all-important to preserve as much normal tissue as possible.

The mode in which Chian turpentine effects the removal of cancer was well illustrated in a case of epithelioma of the vulva. The patient, aged sixty, had been operated upon for cancer of the clitoris or vulva. The case was reported in the *Lancet* at the time. The patient thoroughly recovered from the operation. About a year afterwards cancer reappeared in the lower part of the vulva and vagina. The growth was again excised, and she made a good recovery this time. About eight months afterwards a cancer appeared on the right labium, and when it was there seen for the first time it was the size of half-a-crown. It was determined to give the Chian turpentine, and the drug was given in full doses three times daily. At the end of the first week there was no alteration, excepting that the cancer was thought to be somewhat paler on its surface, and the surrounding swelling less. Second week: The growth was coated with a secretion of a grayish color, which appeared to be firmly adherent. Fourth week:- The growth was only half its former size. Its surface was somewhat convex; and was considerably thicker than previously, and was surrounded by a ring of a bright crimson color. One of the resident surgeons of the hospital who saw it remarked that it looked like a small mushroom springing from the vulva. Sixth week: The growth was now diminished to the size of a fourpenny piece, and was still surrounded by the bright colored ring, but was free from the secretion. The surface did not bleed on being touched. Eighth week: The colored ring had disappeared. The growth was much smaller, and it had the appearance of a large "seedy" wart, such as is sometimes seen on the hands. Tenth week: The growth remained about the same. Twelfth week: The growth had all but disappeared, and the patient went into the country for a short time, but promised to report herself when she returned.

In private practice three cases of cancer of the rectum are under treatment, two of which are much improved; and one has not been seen again, but a favorable report is given after seven weeks' trial. Three cases of cancer of the stomach are under treatment; one has discharged herself, but the other two have remarkably improved, as regards freedom from pain and capacity of retaining nourishment, though continual sickness existed previously. So far as the treatment has extended, these cases seem to prove that the turpentine is equally, if not more, efficient in cancer of the stomache than any other organ. In one case the glands of the neck were much enlarged; but these swellings entirely disappeared after the use of the remedy. Four cases of cancer of the tongue have been under observation. In one case the growth was as large as a cherry, and in four weeks it was reduced to a level with the mucous membrane, and it appears now to be cicatrizing.

Several cases of cancer of the face have been very much benefited. In two cases of endothelial cancer of the abdomen and one of cancer of the liver, no benefit seemed to be derived, excepting that in one case there was some alleviation of pain, and an improvement of the general health for a time.

In all forms of cancer when the turpentine has been taken for six or eight weeks, I have found the administration of a tonic to be of advantage, as it promotes the convalescence of the patient, and if the disease affects the uterus or rectum it seems to facilitate the escape of the debris from the destroyed mass, and to promote cicatrization. Four minims of the tincture of perchloride of iron, and three minims of liquor strychniæ, taken three times daily, are recommended; and if there be any pain, which often happens after six months' use of the remedy, six drops of tincture of belladonna are added. A generous diet is allowed. Morphia is not advised. The bowels should be kept daily moved, when necessary, by a mild aperient. After the turpentine has been regularly taken for three or four months, I now recommend it to be discontinued for three or four days in the fortnight. It is probable that future experience will show that, after it has been taken for the above named period, it will be no longer necessary in the majority of cases, but as a precautionary measure it has been generally continued in the manner just described.

There are sixteen cases of cancer of the breast under treatment in the hospital, and a large number are under observation privately. The effects of the remedy in the first few weeks are, the relief of pain, and a diminution in the size of the breast, which is generally more movable, even when apparently fixed to the walls of the chest; this probably rises from a diminution of the surrounding cancerous infiltration. Time has not been sufficient, however, to speak with definiteness as to the ultimate results. The clinical facts brought out in this class of cases are very interesting and instructive. There is a great probability that the disease is arrested, as after four months' trial of the remedy the morbid growth has not increased in size. As far as can be stated at present, for therapeutical purposes the cases appear to be divided into three classes: 1. The ordinary scirrhus of the breast, with or without surrounding infi tration. 2. Epithelial cancer. 3. Cases of ulceration after previous operation.

In the *first* class of cases great interest attaches to the experiment, as indicating how the solid mass is removed. It appears at present to deport itself as if it were a foreign body, and seems disposed to make its way rapidly to the surface; but in the cases where this has occurred the growth is not of an active character, for as the mass comes to the surface a crust forms upon it. This disappears in time, and another forms, while the whole bulk of the mass gradually diminishes in size. It may prove eventually that this is the one mode of the removal of the disease. In these cases the growth does not increase in size, but, on the contrary, gradually diminishes.

In the *second* class —*i. e.*, epithelial cancers—the surface of the growth becomes paler at first, then smoother, as if it had been levelled by friction ; sometimes some of the nodules slough away. Then a process of cicatrization takes place. There are two cases in which the new tissue around the growth is quite half an inch in breadth. Two others, about one and a half or two inches respectively in diameter, have entirely healed over.

In the *third* class of cases the results are so far favorable that the ulceration has not increased in size, but they have not progressed so favorably as the other cases. In several instances the glands of the axilla have been enlarged, but the enlargement has generally disappeared under a steady use of the turpentine.

The importance of these imperfect notes to the profession, and the influence they may have in aiding the treatment of cancer will, I hope, be a sufficient apology for reverting to the subject. At least I trust I have shown that the remedy is not useless. I may, in conclusion, state that in the treatment of these diseases patience and perseverance for a few months are absolutely necessary. In some diseases which affect the human race a continued treatment for twelve or eighteen months is often thought requisite. It has, I believe, been shown that the remedy does produce some influence on the progress of the cancer ; and it is but reasonable to require that a similar indulgence be extended to the treatment of the cancer by this method, as in the chronic diseases just referred to. As the remedy can be taken with impunity for a long period, and if time be granted for the trial of it I feel that I am justified in stating that its steady use promises excellent future results.

